# Development of an infection control competency scale for clinical nurses: an instrument design study

**DOI:** 10.1186/s12912-024-01904-1

**Published:** 2024-04-19

**Authors:** Yong Hwan Hyeon, Kyoung Ja Moon

**Affiliations:** College of Nursing, Keimyung University, 1095 Dalgubeol-daero, 42601 Daegu, South Korea

**Keywords:** Infection control, Concept analysis, Exploratory factor analysis, Content validity index, Reliability

## Abstract

**Background:**

Nurses work in close proximity to patients, and as such, they can have a direct impact on the control of infections; thus, it is important for nurses to be competent in infection control. However, the scales used to measure infection control performance in nurses are not suitable for measuring infection control competencies that reflect nurses’ expertise, clinical environment, and work. Thus, this study aimed to develop a valid and reliable measure to assess infection control competency of clinical nurses.

**Methods:**

A concept analysis, using a hybrid model, was performed on the infection control competency of clinical nurses to confirm the components and develop 67 initial items. Ten experts evaluated the content validity of these items, and a Korean language expert and a Doctor of Nursing reviewed the questions to consolidate them into 59 items. Subsequently, 267 nurses working at a certified tertiary hospital in D City were surveyed to confirm the validity and reliability of the scale.

**Results:**

As a result of the study, the final scale comprising seven factors and 33 questions was derived, and the cumulative explanatory power of these factors was 60.8%. To verify convergent and discriminant validity, confirmatory factor analysis was conducted, and the average variance extraction index, composite reliability values, and confidence interval of the correlation coefficient between factors were confirmed. Convergent and discriminant validities were verified by comparison with standard values. The Cronbach’s α for the entire scale in this study was 0.93. Consequently, the validity and reliability of the clinical nurses’ infection control competency measurement scale were verified.

**Conclusions:**

The validity and reliability of the infection control competency measurement scale for clinical nurses (ICCS-CN) developed in this study was verified, and the scale can be effectively used to measure the infection control competency of clinical nurses. Measuring the infection control competency of clinical nurses will help reduce the harm caused by infection and ensure patient safety by decreasing infection rates in medical institutions.

**Supplementary Information:**

The online version contains supplementary material available at 10.1186/s12912-024-01904-1.

## Background

Infection control is defined as minimizing the acquisition and spread of an infectious agent [1] and is performed to prevent infection or spread in a healthcare facility [2]. Infections occurring in medical institutions cause socioeconomic losses due to increased hospitalisation periods, medical expenses, and medical disputes. Infection is a major cause of death and poses a serious threat to patient safety [3]. Therefore, the importance of infection control has been emphasised to prevent the harm caused by infection and protect patient safety.

Nurses are medical personnel who work closely with patients; therefore, they can directly impact infection control [4]. To this end, nurses must thoroughly comply with infection control guidelines, be equipped with the latest knowledge and skills for infection control, and play a key role in infection control, such as assessment of patients’ infection symptoms and providing education [5, 6]. However, in previous studies on nurses, the strict practice of infection control guidelines, including hand hygiene, was low [7, 8]. In particular, sometimes old guidelines are applied or infection control guidelines are simply not followed, even though nurses are aware of them [9]. Additionally, nurses did not know much about multidrug-resistant bacteria, did not participate in the antibiotic use process, and did not provide sufficient education to those patient to isolate [9]. Overall, infection control practices appear to be limited.

Infection control competency is defined as the integration of knowledge, skills, and behaviours for performance of infection control in accordance with infection control standards, and it consists of competencies such as basic microbiology, hand hygiene, education, communication, and critical thinking [10–12]. Infection control capacity helps medical professionals to quickly apply new guidelines and supports the optimal use of antibiotics [12]. It can also contribute to reduction of infection rates by enabling the application of the best evidence in clinical settings where knowledge and skills are constantly changing [13]. Therefore, it is important for nurses to possess infection control competencies to protect themselves and patients from infections [14].

Yu et al. [15] reported on their scale developed a scale for measurement of neonatal intensive care unit nurses’ infection control competency. However, to accurately measure a nurse’s competency, it is necessary to reflect on the situation related to the patient, clinical environment, and nurse’s work [16]. The scale developed by Yu et al. [15] focussed on the neonatal intensive care unit environment, which has limitations in terms of measuring the infection control competency of nurses caring for patients compared to other environments. In addition, studies related to infection control competency include that by Carrico et al. [17], which presented a list of infection control competencies of healthcare personnel, and another by Liu et al. [10], which presented a list of infection control competencies for new nurses. However, Carrico et al. [17] developed a general infection control competency list for all medical personnel, which did not present nurses’ infection control competencies specifically. In addition, Liu et al. [10] focused on hand hygiene, personal protective equipment, personal safety, standard precautions, and attention to transmission routes. Although cooperation, communication, and leadership are suggested as important attributes of nurses’ infection control competency [6, 11], they were not clearly revealed in the study by Liu et al. [10]. In addition, these infection control competency list development studies were conducted to present a list and a basic framework for infection control competency [10, 17]. The list of infection control competencies mentioned has been developed not only to encompass professions other than nurses or to focus on newly graduated nurses but also to be based on expert consensus. The reliability and validity verification procedures for using it as a tool have not been conducted, and it consists of an excessive number of questions for use as a scale.

Therefore, in this study, the attributes of the infection control competency of clinical nurses were derived through concept analysis using the hybrid models of Schwartz-Barcott and Kim [18]. A scale was developed to measure the infection control competencies of clinical nurses in accordance with the scale development guidelines of DeVellis [19] and we aimed to verify the validity and reliability of the scale.

## Methods

This methodological study developed a clinical nurse’s infection control competency measurement scale and tested the validity and reliability of the instrument. It was conducted according to the scale development guidelines presented by DeVellis [19].

### Step 1. Confirming the infection control competency attributes of clinical nurses

To confirm the components of the scale, a concept analysis method using a hybrid model was applied [18] to confirm the infection control competency attributes of clinical nurses. In the theoretical stage of the concept analysis, a list of infection control competencies and literature related to infection control competencies were reviewed. We were able to confirm the original text and conducted a search for literature written in either Korean or English. We entered keyword combinations into search engines and conducted literature searches and did not impose any restrictions on the search initial period to minimize bias in the data.

1. Search period: No restrictions ~ January, 2022.
2. Databases: Pubmed, CINAHL, RISS, KCI, DB pia.
3. Keywords: ‘infection control,’ ‘competency,’ ‘competence,’ ‘clinical competence,’ ‘competency assessment,’ ‘nurses’ (combination example in Pubmed: ((“Infection Control”[Mesh]) AND “Nurses”[Mesh]) NOT “Infection Control Practitioners”[Mesh], “infection control” AND competen* NOT link, etc.)
4. Exclusion criteria: Cases focusing solely on specific occupational groups such as infection control specialist nurses, nursing students, and stoma specialist nurses. Papers that have low relevance to the research topic and those that are duplicates were excluded.
5. Additionally, we reviewed infection control guidelines and infection control-related research through hand-searching, and used the references from this literature into attribute derivation.

In the fieldwork stage, 12 nurses with more than 7 years of clinical experience participated in the interviews. All interviews were recorded, transcribed, and analysed by marking the important or meaningful parts. Based on the anticipated ability to describe rich experiences in infection control and attributes of infection control competencies, nurses with over 7 years of experience at a tertiary hospital in Region D were selected as participants for the fieldwork phase. The interviews were conducted from December 8, 2021, to December 19, 2021, lasting between 96 and 115 min.

The main questions included: ‘What do you think infection control competencies are, and what are their components?’, ‘If you have any experiences regarding the importance of infection control, please share’, ’What are the ways to enhance infection control competencies?’, ‘Please share your experiences in performing infection control while caring for immunocompromised or cancer patients’, ‘When did you feel that infection control was important?’, ‘In relation to the outbreak of novel infectious diseases, what infection control competencies do you think nurses should have?’, ‘What do you think are the competencies that nurses need for infection control?’, ‘Do you have any exemplary cases of nurses with infection control competencies in the nursing field?’, and ‘Are there any cases in the nursing field where nurses lack infection control competencies?’.

These questions were implemented until no new information regarding infection control competencies emerged. The attributes of infection control competencies for primitive clinical nurses were derived by analysing the attributes identified through theoretical and fieldwork stages.

### Step 2. Item generation

The items were developed based on the attributes and sub-attributes that appeared during the concept analysis stage. Efforts were made during the item generation process to faithfully incorporate the realities of infection management, reflecting the actual situation of infection control. For this purpose, existing studies on the role of nurses in the infection control process were used. Additionally, statements collected directly from participants through interviews at the site were also reviewed and integrated to develop the items.

### Step 3. Face validity test

In the item review stage, a small group of five nurses from a tertiary general hospital and two doctoral students in nursing checked items such as readability, expression, and understanding of the words used in the initial items developed.

### Step 4. Content validity test

The concept of clinical nurses’ infection control competency and the purpose of the study were explained to a group of experts, and content validity was tested by examining the content validity index (CVI). The criterion for CVI was ≥ 0.90 [20]. The expert panel consisted of 10 members, including one medical doctor, three nursing science doctors, and six nurses with over 10 years of clinical experience.

### Step 5. Field test

#### Sample

The recommended sample size for the verification of the measurement scale was at least 200 [21] or five times the number of items [22]. In this study, 324 participants were included to account for a dropout rate of 10%, based on 5 out of 59 items passing content validity. A total of 283 questionnaires were collected and 267 were utilised for statistical analysis after excluding inappropriate questionnaires (Doubtful questionnaires were excluded, such as those that did not meet the research subject criteria, questionnaires with only one score, and those in which not all items were filled out). Although this fell slightly short of five times the number, it still satisfied the minimum criterion of 200 or more.

#### Data collection

A survey was conducted with nurses who had worked for more than 6 months at a tertiary general hospital in region D, and the time required to respond to the survey, understanding of the overall questions, and opinions on corrections were collected. A notice about the study was distributed to each department, and nurses participated voluntarily. The selection criteria for the survey comprised the provision of direct care to patients, an understanding of the purpose of the study, and the agreement to participate. The exclusion criteria were: (i) nurse manager, (ii) less than 6 months in the nursing profession; (iii) currently in the orientation period; and (iv) working in diagnosis and treatment departments (endoscopy room, rehabilitation treatment room, artificial kidney room, radiology department), anaesthesia and recovery rooms, delivery rooms, operating rooms, or central supply rooms within special departments.

### Step 6. Evaluate the scale

Data collected for the validity and reliability testing of the measurement scale developed in this study were analysed using SPSS/WIN 27.0 and AMOS 27.0.

#### Construct validity

The construct validity of the items was confirmed through an exploratory factor analysis (EFA). In the item analysis, the mean, standard deviation, kurtosis, and skewness were confirmed. The EFA method used principal component analysis and Equamax rotation; the number of factors was fixed at seven according to the factors derived in the concept analysis stage. The suitability of the data for EFA was checked using Bartlett’s test of sphericity and the Kaiser-Meyer-Olkin (KMO) value, with a significance level (*P* <.05) and a KMO value of ≥ 0.80 as a prerequisite, and all common factors must explain at least 40% of the total variance [23].

To confirm the contribution of the question, the correlation between the question and the total score was analyzed, and deletion of the question was considered if the correlation coefficient was less than 0.30 [24]. In EFA, commonality criteria was less than 0.40 [25], and the factor loading value of the questions was considered to be less than 0.30 as the deletion criteria [26].

A confirmatory factor analysis was performed to test convergent and discriminant validity, and the average variance extracted (AVE) index ≥ 0.5, composite reliability (CR) values ≥ 0.7 [27], correlation coefficients < 0.85 [28], and confidence intervals between the factors were confirmed. The confidence interval of the correlation coefficient must not include 1 to be considered to have discriminant validity [29].

The criteria used for the fit indices included Normed χ2 (CMIN/df) ≤ 3, Comparative Fit Index (CFI) ≥ 0.90, Tucker–Lewis Index (TLI) ≥ 0.90, Root Mean Square Error of Approximation (RMSEA) ≤ 0.05, and Standardized Root Mean-square Residual (SRMR) ≤ 0.08 [30–32].

#### Reliability

The internal consistency and reliability of the scale was checked using Cronbach’s alpha (α) for the entire scale and for each factor. Cronbach’s alpha was deemed reliable when it more than 0.6 [33].

## Results

### Confirming the infection control competency attributes of clinical nurses

A total of 15,582 documents were reviewed. Among the retrieved documents, those focusing on professions other than nurses, such as infection control nurse, nursing students, physical therapists, so on and studies with low relevance to the research topic (e.g. fragmented infection control performance measurement studies), were excluded. Among the searched papers, those dealing with concept definitions, attributes, roles, so on, that related to infection management by nurses were selected for final analysis, and a total of 12 documents were ultimately reviewed. Through the literature review, there eight attributes were derived from the theoretical stage, including basic microbiology, assessment of infection symptoms, standard precautions and transmission-based precaution, leadership, critical thinking, risk and emergency preparedness, communication, and education.

Qualitative data were collected through interviews stage of fieldwork. It was confirmed that the properties derived by analyzing the content collected through individual and group interviews were similar to the properties derived through the theoretical stage. There were seven attributes derived at the field stage: basic microbiology, clinical assessment and risk assessment, standard precautions transmission-based precaution, leadership, critical thinking, communication, and education.

The attributes and their contents from the theoretical and fieldwork stages were analysed to derive primitive attributes. The review process involved comparing the attributes and contents derived from the theoretical and fieldwork stages of the respective concept, assessing the practical applicability and significance, determining the validity of the initial selection of the concept, and comprehensively verifying whether various research findings support the concept. The seven derived primitive attributes and their sub-attributes are shown in Table S1.

### Item generation

Sixty-seven initial items were developed by referring to infection control guidelines, deriving attributes and sub-content. The number of items per attribute as follows: basic microbiology 7 items, infection risk assessment 5 items, infection control practice 16 items, leadership 18 items, critical thinking 11 items, communication 5 items, and education 5 items.

### Initial item face validity and content validity

The face validity of the 67 initial items was assessed based on feedback from a small group comprising five nurses from tertiary hospitals and two nursing doctoral students regarding readability, expression, and comprehensibility, which led to the removal of two items. The scale level, CVI (S-CVI), derived from the expert content validity test for the 65 items, constructed after concept analysis, was 0.93, which satisfied the standard. By modifying some detailed words and expressions, this process resulted in 59 items.

### Field test

The general characteristics of the 267 participants in the field test indicated that 93.6% were female, and the age group of 26 to 30 years accounted for the largest proportion at 44.2%. Moreover, 83.5% of participants held bachelor’s degrees, and 90.3% of nurses had previous education related to infection control. The participants’ clinical experience showed that 37.5% of participants had more than 7 years of experience (Table 1).

**Table 1:** General characteristics (*n* = 267)

Characteristics	Categories	n(%)
Gender	Male	17 (6.4)
	Femle	250 (93.6)
Age(years)	Less than 26	61 (22.8)
	26 ~ 30	118 (44.2)
	31 ~ 35	60 (22.5)
	36 ~ 40	24 (9.0)
	More than 41	4 (1.5)
Education	Diploma	28 (10.5)
	Bachelor’s degree	223 (83.5)
	Master’s degree	15 (5.6)
	PhD	1 (0.4)
Attending infection control education	Yes	241 (90.3)
	No	26 (9.7)
Career(years)	0.5 ~ 1	46 (17.2)
	2 ~ 3	58 (21.7)
	4 ~ 6	63 (23.6)
	More than 7	100 (37.5)
Experience in infection control roles and responsibilities	Yes	32 (12.0)
	No	235 (88.0)

### Construct validity

#### EFA

To confirm the structure of the concept of infection control competency in clinical nurses, EFA was performed using principal component analysis and orthogonal rotation. Through item analysis before EFA implementation, the average, standard deviation, skewness, and kurtosis of items, and Cronbach’s α value and item-total score correlation were checked to confirm outliers, normality, and contribution for 59 items.

Item analysis, the average of each item was 2.78–4.37, and the standard deviation was 0.51–1.12, showing no extreme values. The skewness was − 0.01– -1.21, and kurtosis − 0.01–2.80, of which the kurtosis was 2.80 for item 50, ‘I provide hospital staff (e.g. patient transport staff, cleaning staff, blood collection team, transfusion team) visiting patients with information about infection transmission pathways and relevant infection control measures that should be observed in relation to the patient’ was removed, the Cronbach’s α value for the 58 items was 0.95, and no items lowered the Cronbach’s α value.

After checking the item-total correlation values of 58 items to confirm item contribution, the items with item-total correlation coefficients less than 0.30 were item 31 ‘My contact can be the cause if multidrug-resistant bacteria spread to the people I care for’, item 38 ‘When I see a nurse who is actively involved in infection control, I feel unusual [reverse question]’, and item 39 ‘I think that when an infection control problem occurs in a department, the first priority is to find the employee who caused the problem [reverse question]’. These items were removed because it was judged that they would pose difficulty in attaining an appropriate response and the contribution of these item was low because they were related to social desirability or consisted of reverse questions.

To test the construct validity of the 55 items derived after item analysis, five sessions of EFAs were conducted to confirm the Kaiser–Meyer–Olkin (KMO) value, Bartlett’s sphericity test value, factor structure, and loading.

As a result of the EFA for the first 55 items, the KMO value was 0.93 and the Bartlett’s sphericity test value was *χ²* = 7445.48, df = 1540, *p* <.001, which was appropriate for factor analysis.

For the first round of exploratory factor analysis of 55 items, the Kaiser-Meyer-Olkin (KMO) measure of sampling adequacy was 0.93, and Bartlett’s test of sphericity yielded χ²=7445.48, df = 1540, *p* <.001, indicating the appropriateness of conducting the factor analysis. The cumulative explained variance of the factors was 51.0%. However, items 27, 28, 32, 35, 42, and 51 showed communalities below 0.4 (specifically 0.33, 0.38, 0.34, 0.39, 0.39 respectively), prompting the removal of these six items before conducting next factor analysis.

For the second round with 49 items, KMO value was 0.92, and Bartlett’s test yielded χ²=6338.11, df = 1176, *p* <.001, indicating suitability for factor analysis. The cumulative explained variance of the factors was 53.6%. However, items 22 and 25 exhibited communalities below 0.4 (specifically 0.37 and 0.39 respectively), leading to the removal of these two items before factor analysis.

In the third round, with 47 items, the KMO value was 0.92, and Bartlett’s test yielded χ²=6137.52, df = 1081, *p* <.001, indicating suitability for factor analysis. The cumulative explained variance of the factors was 54.9%, with communalities ranging from 0.43 to 0.72, meeting the criterion for communalities. However, there were 12 items that did not converge with factors derived from the concept analysis stage or other items.

In the fourth round with 35 items, KMO value was 0.91, and Bartlett’s test yielded χ²=4274.520, df = 595, *p* <.001, indicating suitability for factor analysis. The cumulative explained variance of the factors was 59.6%, with communalities ranging from 0.46 to 0.76, meeting the criterion for communalities. As a result, item 7, ‘I observe areas vulnerable to infection in patients (e.g., surgical and wound sites, insertion sites for invasive devices or drainage tubes) during each shift’, was deemed related to patient conditions and was deleted. Item 17, ‘I determine the visiting order considering isolated and reverse isolated patients during patient rounding’, although related to infection control practices, was deleted as it loaded with items related to patient education factors.

For the fifth round, with 33 items, the KMO value was 0.91, and Bartlett’s test yielded χ²=4022.756, df = 528, *p* <.001, indicating suitability for factor analysis. The cumulative explained variance of the seven factors was 60.8%, and communalities ranged from 0.45 to 0.76, meeting the criterion for communalities.

As a result of the final EFA of 33 items in the 5th order, the KMO value was 0.91 and the Bartlett’s sphericity test value was *χ²* = 4022.756, df = 528, *p* <.001, which was appropriate for factor analysis. For the seven factors derived as a result of the EFA, the factors were named to represent all items belonging to each factor. The seven factors are: (1) Basic Microbiology (2) Critical Thinking (3) Communication and Patient Assessment (4) Compliance with Infection Control Guidelines (5) Education of Patient (6) Infection Control Leadership (7) Prevention of Occupational Exposure.

The cumulative explanatory power of the seven factors was 60.8%, and the commonality was 0.45–0.76, confirming that the criteria for commonality were satisfied. As a result of the final EFA, 33 items and seven factors appeared, and the overall explanatory power was 60.8%. The explanatory power of factor 1, 2, 3, 4, 5, 6, and 7 was 9.9%, 9.9%, 8.9%, 8.5%, 8.2%, 7.9%, and 7.9%, respectively. The average explanatory power was 7.3% and the commonality ranged from 0.46 to 0.77 (Table S2).

### Confirmatory factor analysis and item convergent-discriminant validity

To assess the model fit of the infection control competency tool for clinical nurses, a CFA was conducted, consisting of seven factors derived from the EFA. In the initial model, the partial fit indices were not ideally satisfactory (RMSEA was 0.056 (90% confidence interval: 0.051 to 0.062, p-close 0.035), SRMR was 0.060, TLI was 0.878, and CFI was 0.891). Therefore, we reviewed the modification index and conducted CFA again based on it. The modified model fit indices of the measurement model inputted into CFA were as follows: RMSEA was 0.052 (90% confidence interval: 0.046 to 0.058, p-close 0.309), SRMR was 0.056, TLI was 0.898, and CFI was 0.909 (Table 2). We found that the generated model was appropriately derived from theory and exhibited TLI and CFI values approximating the cutoff, along with optimal RMSEA and SRMR values, we adopted this model.

**Table 2:** Model fit indices for infection control competence of clinical nurses (*n* = 267)

Model	CMIN/df (≤ 3)	RMSEA (≤ 0.05)	LO	HI	PCLOSE	SRMR (≤ 0.08)	TLI (≥ 0.90)	CFI (≥ 0.90)
Initial model	1.850	0.056	0.051	0.062	0.035	0.060	0.878	0.891
Modified model	1.713	0.052	0.046	0.058	0.309	0.056	0.898	0.909

As a result of the CFA to verify the convergence validity of the measurement scale the AVE value of the scale was 0.53–0.69 and the CR value was 0.78–0.90. The criteria were met, and convergent validity was secured. The discriminant validity of the measurement scale was tested in two ways. First, it was confirmed that the value of the correlation coefficient(r) between factors was 0.37–0.83, which did not exceed 0.85 (Fig. S1).

Second, discriminant validity was secured because 1.0 was not included in the confidence interval of the correlation coefficient between the factors derived through the CFA (Table S3).

### Reliability

To test the internal consistency reliability of ICCS-CN(Appendix A) developed in this study, the Cronbach’s α value of all items and each factor, and the correlation coefficient between the items and the total score were checked. The Cronbach’s α value of all items was 0.93, and the Cronbach’s α value of each factor was 0.84 for the Basic Microbiology factor, 0.85 for the Critical Thinking factor, 0.83 for the Communication and Patient Assessment factor, 0.77 for the Compliance with Infection Control Guidelines factor, 0.79 for the Education of Patient factor, 0.73 for the Infection Control Leadership factor, and 0.63 for the Prevention of Occupational Exposure factor. The Cronbach’s α value of all factors was over 0.60, which satisfied the reliability criterion for internal consistency (Table 3). The item-total score correlation coefficient was 0.39–0.67, and the correlation coefficient standard was 0.30 or higher, which satisfied the reliability standard.

**Table 3:** Internal consistency of scales

Factor	Number of questions	Cronbach’s α
1. Basic microbiology	4	0.84
2. Critical thinking	6	0.85
3. Communication and patient assessment	7	0.83
4. Compliance with infection control guidelines	5	0.77
5. Education of patient	4	0.79
6. Infection control leadership	4	0.73
7. Prevention of occupational exposure	3	0.63
Infection control competency of clinical nurses	33	0.93

## Discussion

This study aimed to develop and verify a scale for measuring the infection control competency of clinical nurses. The infection control competency measurement scale for clinical nurses (ICCS-CN) consists of 33 items and seven factors. The first factor, ‘Basic Microbiology’, comprises content pertaining to the interpretation of test results related to the understanding of pathogenic bacteria, and the explanatory power was 9.9%. In a situation where more than 70% of microorganisms associated with infections in medical institutions are related to bacteria, the incidence of multidrug-resistant bacteria is increasing, and patients infected with multidrug-resistant bacteria are reported to have a poorer prognosis than patients infected with non-resistant bacteria [34, 35]. The ‘Basic Microbiology’ factors derived from this study are important factors for nurses to perform infection control in a clinical environment. This content is similar to the basic microbiological properties detailed in the study by Liu et al. [10], which derived a list of new infection control competencies for nurses, thereby supporting their results. For effective infection control, correct knowledge and understanding of microorganisms is required, and knowledge and understanding of basic microorganisms enables optimal antibiotic treatment for patients [36]. Nurses also play an important role in preventing the risk of antibiotic resistance and should ensure that appropriate antibiotics are used [37]. To this end, it is important for nurses to understand the transmission mechanism of microorganisms, to be aware of the chain of infection, and to have the ability to interpret the results of microbial tests and antibiotic susceptibility [11, 38]. Therefore, the ‘Basic Microbiology’ factors were able to measure these important components.

The second factor, ‘Critical Thinking’, includes content that explores and applies the latest credible evidence, reflecting a critical perspective on issues related to infection; it had an explanatory power of 9.9%. Nurses should not practice without utmost care in a specific situation but should analyse information, reason with it, and apply it [39]. In addition, critical thinking in nursing is a characteristic indicator that connects theory and practice, and in infection control situations, the period during which an indwelling catheter is inserted is shortened by applying theory to prevent urinary tract infection in practice [40], thus supporting the results of this study. In other words, for infection control, applying the latest reliable guidelines and literature related to infection control to practice is an important role included in infection control competency [10, 41, 42]. The items constituting the ‘Critical Thinking’ factor reflect this theoretical background effectively.

The third factor, ‘Communication and Patient Assessment’, comprises questions related to the sensitive assessment of the patient’s potential risk and symptoms of infection, and information exchange and free communication between medical staff regarding the assessed patient’s infection information and treatment direction, and the explanatory power was 8.9%. Communication also includes the keeping of detailed records related to infection control, and communication between various hospital staff, including doctors and nurses, is essential for effective infection control, as it increases cooperation and work efficiency and leads to prompt and accurate testing [37, 43]. In addition, communication between medical staff is the process of conveying information about the patient, including collecting and transmitting information about test results and potential risks [44]. This content is sufficiently reflected in the ‘Communication and Patient Assessment’ factors. In addition, the communication factor was not derived from the study by Liu et al. [10], but from the study by Massaroli et al. [11], which suggested communication as a key element of nurses’ infection control competency, thus supporting the results of this study. Therefore, the ‘Communication and Patient Assessment’ factors that could measure communication in clinical infection control situations.

The fourth factor was ‘Compliance with Infection Control Guidelines’, which consists of items such as hand hygiene, application of aseptic methods when administering drugs, and management of isolated patients. The explanatory power of this factor was 8.5%. Hand hygiene, in particular, is an effective and easy way to prevent healthcare-associated infections and reduce the spread of multidrug-resistant bacteria [45]. As nurses often have frequent contact with patients and invasive nursing behaviour, it is important to perform hand hygiene routinely and habitually at a high standard [46]. In addition, compliance with aseptic techniques when administering drugs, along with hand hygiene, are basic elements of infection control [47] and the items derived from this scale are the results of the theoretical importance reflected in the scale.

In addition, the use of exclusive medical supplies with those isolated patients is included in the concept of item management and contact caution for multidrug-resistant bacterial infection control [48]. Contact caution recommendations consist of patient placement, personal protective equipment use, patient movement restrictions, treatment equipment and equipment management, environmental management, and visitor management, and there are exclusive management items for patients in isolate corresponding to treatment equipment and equipment management [47]. Liu et al. [10] suggested hand hygiene, standard precautions, propagation route-specific attention, and personal protective equipment factors, which were similar to those in this study, but these factors were presented separately. The difference is that it was derived by dividing it into the ‘preventing occupational exposure’, ‘compliance with infection control guidelines’ factors, and since the composed items also include items related to personal protective equipment, it can be seen that these items are supported at the overall scale level. In addition, as hand hygiene, wearing of protective equipment when in contact with blood or body fluids, and daily cleaning of the environment are included in internal medical aseptic techniques [47, 49]. ‘Compliance with Infection Control Guidelines’ is a factor that measures an important competency at the practical level of infection control by nurses.

The fifth factor was ‘Education of Patient’, which comprised patients’ education on infection control and behavioural changes, and the explanatory power was 8.2%. Education is a nurse’s professional responsibility and an independent function, which allows patients to manage their own health and maintain their health [50], therefore, it can be said that it is important to provide education to patients to prevent infection [6]. For example, to properly administer antibiotics, the patient must know the type of antibiotic, the reason for administration, and the side effects and related symptoms caused by the antibiotics [51]. This is because it is necessary to immediately report the side effects of antibiotics to medical staff when they occur, and nurses play an important role in educating patients [52].

In addition, it has been reported that inpatients under isolate have high levels of anxiety and depression when sufficient explanations are not provided regarding the sudden progress of isolation or infection, indicating the need for sufficient education, considering the capability of the patient [9]. Also, to prevent infection, the patients themselves must participate in infection control measures [6]. Therefore, the ‘Education of Patient’ factor reflects the professional characteristics of nurses for infection control and recognises the barriers to patient education and behavioural change education and training using various methods.

The sixth factor, ‘Infection Control Leadership’, which consisted of content related to cooperation with hospital staff and self-leadership, had an explanatory power of 7.9%. Leadership is needed not only in senior positions or executives but also for individual nurses, and will drive change for their colleagues and other team members [5, 6]. Additionally, clinical nurses can indirectly become role models for others by self-regulating their behaviours and attitudes [53]. Self-leadership related to one’s own behavioural control has also been shown to affect the performance of standard guidelines [54]. In addition, in clinical practice, there can be resistance from nurses when receiving feedback on infection control from colleagues or infection control nurses [9, 55], but for infection control to be carried out in medical institutions, when a nurse receives feedback from infection control experts or colleagues, they need to modify their behaviour [6, 56]. Therefore, it was confirmed that self-leadership is necessary to improve behaviour by reflecting on appropriate feedback from others and to comply with infection control guidelines; this content was well represented in the questions for infection control leadership factors.

Collaboration with other employees in environmental management also consists of questions on infection control leadership factors. In the clinical environment, surfaces of the hospital room, such as the toilet seat, are important sources of microbial transmission; therefore, thorough environmental management is required, and nurses must achieve an appropriate level of cleanliness through collaboration with environmental management personnel [6]. However, not only environmental management but also infection control requires cooperation between medical staff. If the nurse is unable to resolve it, cooperation between infection control teams may be required [5, 6]. Cooperation between medical staff is considered an important aspect of infection control leadership.

The seventh factor was ‘Prevention of occupational exposure’, and personal protective equipment properties and occupational exposure prevention properties were combined as one factor, and the explanatory power was 7.3%. Nurses are exposed to infections more than other occupational groups, so they need to be especially careful [57]. In particular, it is reported that nurses need to be careful about blood exposure to damaged skin or mucous membranes [58, 59]. It was found that the interview participants in the fieldwork stage of this study also experienced infection through skin wounds. Rebmann and Carrico [60] reflected on this importance and presented a list of nurses’ infection prevention capabilities as an occupational health factor, as done in our study. In addition, a study on the development of a scale to measure nurses’ preventive behaviours against blood-borne infections supported this study by including items to protect one’s own skin wounds, wear protective gear suitable for the type of infection exposure, and take precautions against stabbing accidents [61].

The Cronbach’s α value of all items in this study was 0.93, indicating very good reliability, and the Cronbach’s α value of each factor was also found to be 0.63–0.85, confirming good internal consistency. The Cronbach’s alpha value for the seventh factor is somewhat low, likely due to the small number of items. In this study, both the Cronbach’s α values for the scale and factors were found to be above 0.60, indicating good reliability. However, to confirm the scale’s consistency, it is necessary to establish test-retest reliability, which was not validated in this study. Therefore, further research is needed to verify reliability through future studies.

The difference between previous studies and ours was that the attributes derived by Liu et al. [10] mainly focused on hand hygiene, personal protective equipment, personal safety, standard attention, and propagation route-specific attention. Including all of them and adding the attributes of patient education, patient assessment and communication, and infection control leadership is meaningful in that nurses’ infection control capabilities are further expanded and presented. In addition, Carrico et al. [17] and Liu et al. [10] presented a list of infection control capabilities but derived the attributes of infection control capabilities through concept analysis in the absence of scales to measure the infection control capabilities of clinical nurses. This study is meaningful in that we developed a scale with verified validity and reliability and laid the foundation for conducting quantitative research through the scaling of infection control capabilities.

### Limitations

To test the discriminant validity of the scale, the correlation between factors and the confidence interval of the correlation coefficient between factors were checked, and the criterion was satisfied, thereby ensuring discriminant validity. However, the correlation between the factors ‘Education of Patient’ and ‘Communication and Patient Assessment’ was relatively high at 0.83, because education on the patient and communication with medical staff are similar in the context of conveying information [62]. Statistically, construct validity can be secured by meeting the criterion for discriminant validity; however, it is necessary to be careful when using the scale because high correlations between variables can cause multicollinearity.

To confirm the stability of the scale, test-retest reliability must be ensured; however, this aspect was not verified in this study. Therefore, it is necessary to verify reliability through further studies. Also, as reliability was verified by performing a CFA on the data collected from one group, the scale should be applied to other groups and analysed repeatedly using the collected data.

The ICCS-CN did not include nurses working in diagnosis and treatment departments such as endoscopy rooms and artificial kidney rooms, which perform infection control according to each department (e.g. anaesthesia and recovery room, delivery room, operating room, and central supply room). Therefore, it is necessary to conduct follow-up studies targeting research groups from various departments, including nurses from departments not included in this study, as well as from various countries. Moreover, future research should explore empirical comparisons, such as assessing convergent validity, between various scales developed in different cultural contexts, building upon the studies conducted by Carrico et al. [17] and Liu et al. [10].

## Conclusions

The ICCS-CN developed in this study consists of seven factors and 33 items. These factors are ‘Basic Microbiology’, ‘Critical Thinking’, ‘Communication and Patient Assessment’, ‘Compliance with Infection Control Guidelines’, ‘Education of Patient’, ‘Infection Control Leadership’, and ‘Prevention of Occupational Exposure’. It is a 5-point Likert scale, and the higher the score, the higher the infection control capacity. The ICCS-CN developed in this study measures the level of infection control competency of clinical nurses and can be used as basic data to inform new measures to improve infection control competency. It is expected to have a positive effect on infection control.

### Electronic supplementary material

Below is the link to the electronic supplementary material.

Supplementary Material 1

Supplementary Material 2

## Data Availability

The datasets generated and/or analysed during the current study are not publicly available but are available from the corresponding author on reasonable request.

## Abbreviations

HHS: Health and Human Services
CVI: content validity index
S-CVI: scale level content validity index
EFA: exploratory factor analysis
AVE: index, average variance extracted index
CR: composite reliability
Cronbach’sα: Cronbach’s alpha
KMO: Kaiser–Meyer–Olkin
ICCS-CN: infection control competency measurement scale for clinical nurses
