# Supplementary material for: Development of an infection control competency scale for clinical nurses: an instrument design study
Source: BMC Nurs. 2024 Apr 19;23:250. doi: 10.1186/s12912-024-01904-1 (PMC11027540; doi:10.1186/s12912-024-01904-1)
Supplement: Supplementary file 1 — Supplementary Material 1 [file 12912_2024_1904_MOESM1_ESM.docx]

**Supplementary file: Development of an infection control competency scale for clinical nurses: an instrument design study**

Yong Hwan Hyeon^1^, Kyoung Ja Moon^1^*

^1^College of Nursing, Keimyung University, 1095 Dalgubeol-daero, Daegu 42601, South Korea

*Corresponding author

**Table S1**. Attributes and Sub-Attributes of infection control competencies for clinical nurses

| Attributes | Sub-Attributes |
| --- | --- |
| Basic Microbiology | Understanding of microbial growth and colonization concepts |
|  | Understanding of the chain of infection and disease transmission mechanisms |
|  | Understanding of the pathophysiology and treatment of infections |
|  | Understanding of antibiotic-resistant bacteria |
|  | Interpretation of microbial culture test results |
|  | Understanding of antibiotic susceptibility testing |
| Infection  Risk Assessment | Identification of signs of uncommon infections |
|  | Assessment of individuals vulnerable to infections |
|  | Identification of suspected infection cases |
|  | Sensitive awareness of patient conditions and infectious symptoms |
| Infection Control Practice | Hand hygiene |
|  | Understanding of standard precautions and transmission-based precautions |
|  | Personal protective equipment |
|  | Aseptic techniques |
|  | Cleaning/disinfection/sterilization/reprocessing |
|  | Prevention of occupational exposure |
|  | Environmental management |
|  | Patient placement |
| Leadership | Exemplifying best practices in infection control |
|  | Fostering a positive organizational culture regarding infection control |
|  | Sense of responsibility towards infection control |
|  | Ethics |
|  | Self-leadership |
|  | Collaboration |
| Critical Thinking | Approaching evidence-based practice |
|  | Awareness of current guidelines |
|  | Efforts to apply theory to practice |
|  | Critical thinking regarding customary practices |
| Communication | Reporting and documentation of infection-related matters |
|  | Sharing infection-related information |
|  | Notification of patient isolation information among healthcare team |
| Education | Encouragement of patient education and compliance |
|  | Recognition of barriers to patient education |
|  | Ability to demonstrate and educate |
|  | Providing evidence-based education |

**Table S2**. Pattern Matrix of the Exploratory Factor Analysis (Final Stage) (*n* = 267)

| Factor loading | | | | | | | |
| --- | --- | --- | --- | --- | --- | --- | --- |
|  | 1 | 2 | 3 | 4 | 5 | 6 | 7 |
| Factor 1. Basic Microbiology |  |  |  |  |  |  |  |
| I can describe the results of the patient’s antibiotic susceptibility test | 0.81 | 0.15 | 0.05 | 0.16 | 0.17 | 0.10 | 0.02 |
| I can describe the results of the patient’s microbiological culture test | 0.77 | 0.16 | 0.12 | 0.20 | 0.08 | 0.17 | 0.05 |
| I can describe the concept of multidrug-resistant bacteria (e.g. MRSA, VRE, and CRE) | 0.76 | 0.09 | 0.04 | 0.18 | 0.25 | 0.06 | 0.08 |
| I can describe the differences between normal flora, colonisation, and infection | 0.69 | 0.30 | 0.16 | -0.06 | -0.02 | 0.16 | 0.14 |
| Factor 2. Critical Thinking |  |  |  |  |  |  |  |
| When I gather information about infection, I refer to guidelines or trusted literature from which sources are identified | 0.12 | 0.77 | 0.14 | 0.09 | 0.15 | 0.17 | 0.08 |
| When I refer to the literature related to infection, I refer to the latest literature within 5 years | 0.13 | 0.76 | 0.12 | 0.08 | 0.11 | 0.13 | 0.12 |
| I have no difficulty finding literature related to infection control | 0.22 | 0.73 | 0.12 | -0.06 | 0.17 | 0.00 | 0.15 |
| I personally look for information on the latest infection issues | 0.17 | 0.67 | 0.18 | 0.19 | 0.13 | 0.07 | -0.00 |
| I apply theories or evidence related to infection in infection control practices | 0.27 | 0.51 | 0.29 | 0.05 | 0.27 | 0.08 | 0.23 |
| I use it after knowing the antibacterial range of the antibiotics that are mainly used in our department | 0.34 | 0.37 | 0.29 | 0.23 | 0.07 | 0.00 | 0.32 |
| Factor 3. Communication and Patient Assessment |  |  |  |  |  |  |  |
| I freely discuss matters related to the patient’s infection with other medical staff (nurses, doctors, etc.) (e.g. testing, risk of infection, direction of treatment, care) | 0.00 | 0.09 | 0.68 | 0.07 | 0.27 | 0.24 | 0.04 |
| When assessing a patient’s symptoms of infection, I considered uncommon symptoms of infection (e.g. proteinuria, confusion in elderly patients) | 0.17 | 0.25 | 0.64 | 0.09 | 0.14 | -0.02 | 0.27 |
| I record in detail the patient’s risk of infection, symptoms, reports, actions, and changes | 0.10 | 0.13 | 0.56 | 0.16 | 0.36 | 0.09 | 0.06 |
| I share data and information related to infection control with department members | 0.06 | 0.28 | 0.56 | 0.09 | 0.35 | 0.21 | 0.03 |
| I can give my opinion on the re-evaluation of antibiotic administration based on the patient’s test results or theoretical content | 0.38 | 0.27 | 0.47 | 0.09 | 0.27 | 0.11 | 0.13 |
| I notice symptoms of infection or changes in the infection condition that the patient did not have before | 0.24 | 0.16 | 0.46 | 0.36 | 0.06 | -0.00 | 0.34 |
| I consider potential risk factors when assessing a patient’s risk of infection | 0.21 | 0.16 | 0.42 | 0.35 | -0.04 | 0.32 | 0.23 |
| Factor 4. Compliance with Infection Control Guidelines |  |  |  |  |  |  |  |
| It is routine and natural for me to carry out hand hygiene during nursing work | 0.14 | 0.05 | 0.05 | 0.79 | 0.09 | 0.26 | 0.00 |
| I perform hand hygiene in the right way and at the right time | 0.15 | 0.07 | 0.19 | 0.70 | 0.19 | 0.24 | 0.09 |
| When I inject the medicine, I inject the medicine after disinfecting the hub according to infection control guidelines | 0.11 | 0.10 | 0.15 | 0.64 | 0.06 | 0.21 | 0.16 |
| Except for unavoidable cases, I use the medical supplies of the isolated patient exclusively for that specific patient | 0.03 | -0.02 | -0.11 | 0.52 | 0.36 | -0.02 | 0.36 |
| If I find a contaminated place, I don’t neglect it, and I get rid of it as soon as possible | -0.05 | 0.02 | 0.14 | 0.43 | 0.02 | 0.39 | 0.42 |
| Factor 5. Education of Patient |  |  |  |  |  |  |  |
| When I provide infection-related education to the patient, I consider the age, education level, and situation of the patient (e.g. selecting appropriate terms, providing educational materials such as videos and documents, detailed explanations, etc.) | 0.06 | 0.19 | 0.31 | 0.19 | 0.65 | 0.19 | 0.06 |
| I can lead the patient to follow infection control precautions (e.g. personal hygiene, coughing after surgery, hand hygiene, adherence to isolation guidelines, etc.) | 0.03 | 0.12 | 0.21 | 0.14 | 0.64 | 0.17 | 0.22 |
| I understand the psychological state of patients infected with multidrug-resistant bacteria and help them make rational decisions (e.g. administration of antibiotics and cooperation in entering the isolation room) | 0.23 | 0.21 | 0.10 | 0.10 | 0.61 | 0.11 | 0.26 |
| I explain disease information, treatment procedures, and precautions based on evidence when a patient is infected | 0.29 | 0.22 | 0.26 | 0.04 | 0.57 | 0.15 | 0.11 |
| Factor 6. Infection Control Leadership |  |  |  |  |  |  |  |
| I try to improve my behaviour when receiving reasonable criticisms related to infection control from others | 0.00 | 0.09 | -0.09 | 0.25 | 0.22 | 0.67 | 0.06 |
| I try to abide by the guidelines even in situations where infection control is difficult | 0.14 | 0.10 | 0.14 | 0.15 | 0.10 | 0.67 | 0.21 |
| I can request work necessary for infection control from the person in charge of environmental management, such as a cleaning agent | 0.22 | 0.03 | 0.29 | 0.14 | -0.01 | 0.65 | 0.09 |
| strive to improve my weaknesses in knowledge, skills, and practices related to infection control | 0.11 | 0.09 | 0.03 | 0.17 | 0.34 | 0.57 | 0.18 |
| Factor 7. Prevention of Occupational Exposure |  |  |  |  |  |  |  |
| I take steps to protect my skin wounds from infection | -0.00 | 0.07 | 0.10 | 0.08 | 0.10 | 0.04 | 0.81 |
| I wear appropriate personal protective equipment and/or use tools when handling medical waste (e.g. not pressing down waste with feet, using tongs when handling sharps waste, etc.) | 0.06 | 0.14 | -0.01 | 0.03 | 0.13 | 0.28 | 0.71 |
| I wear personal protective equipment appropriate to the expected exposure type and infection transmission route when caring for the patient | 0.18 | -0.10 | 0.20 | 0.20 | 0.31 | 0.26 | 0.39 |
| Eigenvalue | 3.27 | 3.26 | 2.94 | 2.81 | 2.73 | 2.63 | 2.42 |
| Cumulative explanatory power | 9.9% | 19.8% | 28.7% | 37.2% | 45.5% | 53.4% | 60.8% |
| Kaiser–Meyer–Olkin = 0.91; Bartlett’s sphericity test: *χ²* = 4022.756, df = 528, *p* < 0.001 | | | | | | | |


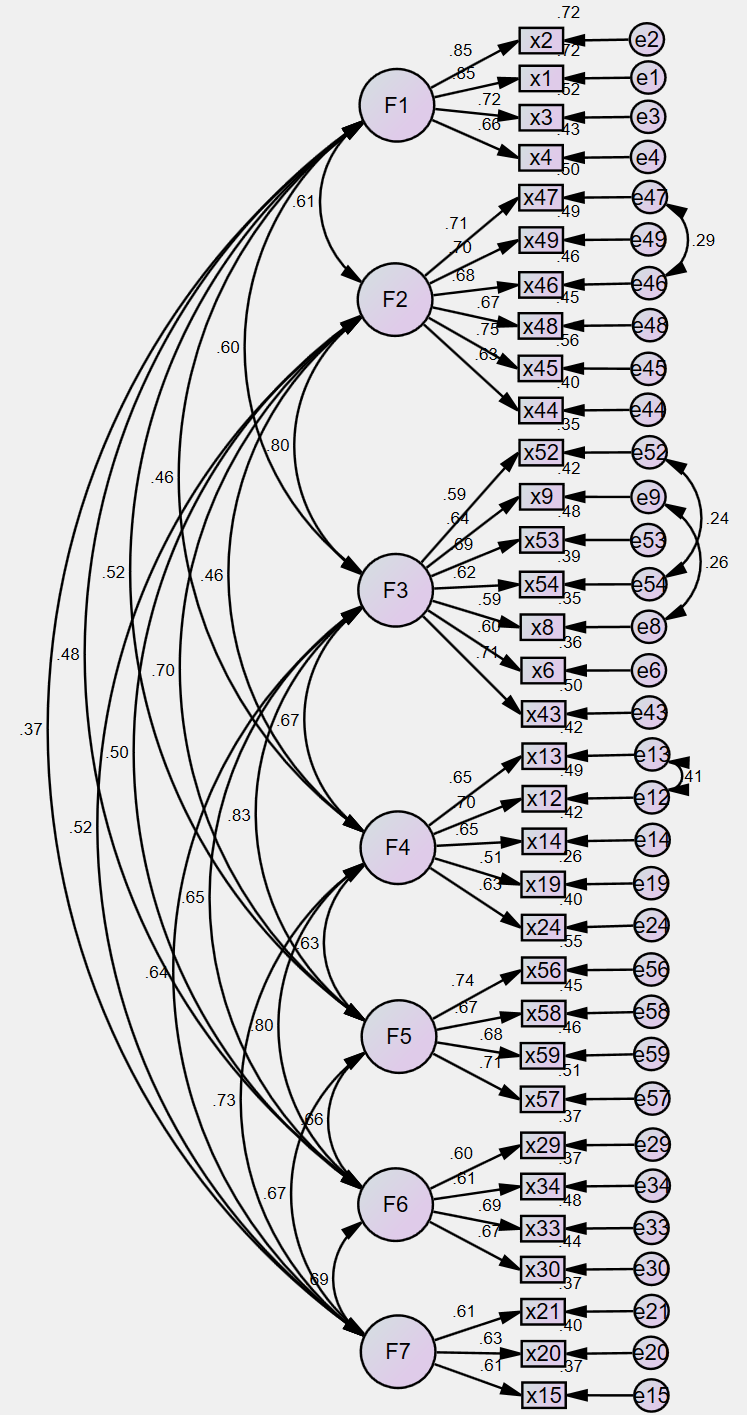


**Figure S1**: Confirmatory Factor Analysis Results

F1: Factor 1, Basic Microbiology; F2: Factor 2, Critical Thinking; F3: Factor 3, Communication and Patient Assessment; F4: Factor 4, Compliance with Infection Control Guidelines; F5: Factor 5, Education of Patient; F6: Factor 6, Infection Control Leadership; F7: Factor 7, Prevention of Occupational Exposure.

**Table S3**. Item Convergent-Discriminant Validity (*n* = 267)

| Factor | Item | FL | SE | AVE | CR | r ± 2SE | | | |
| --- | --- | --- | --- | --- | --- | --- | --- | --- | --- |
| F1 | Item 2 | 0.85 | 0.20 | 0.69 | 0.90 |  | r | SE | ± 2SE |
|  | Item 1 | 0.85 | 0.18 |  |  | F1–F2 | 0.61 | 0.04 | 0.53–0.69 |
|  | Item 3 | 0.72 | 0.29 |  |  | F1–F3 | 0.60 | 0.03 | 0.54–0.66 |
|  | Item 4 | 0.66 | 0.43 |  |  | F1–F4 | 0.46 | 0.03 | 0.4–0.52 |
|  |  |  |  |  |  | F1–F5 | 0.52 | 0.03 | 0.46–0.58 |
|  |  |  |  |  |  | F1–F6 | 0.48 | 0.02 | 0.44–0.52 |
|  |  |  |  |  |  | F1–F7 | 0.37 | 0.03 | 0.31–0.43 |
| F2 | Item 47 | 0.71 | 0.43 | 0.53 | 0.87 | F2–F3 | 0.80 | 0.04 | 0.72–0.88 |
|  | Item 49 | 0.70 | 0.50 |  |  | F2–F4 | 0.46 | 0.03 | 0.4–0.52 |
|  | Item 46 | 0.68 | 0.46 |  |  | F2–F5 | 0.70 | 0.03 | 0.64–0.76 |
|  | Item 48 | 0.67 | 0.49 |  |  | F2–F6 | 0.50 | 0.02 | 0.46–0.54 |
|  | Item 45 | 0.75 | 0.27 |  |  | F2–F7 | 0.52 | 0.03 | 0.46–0.58 |
|  | Item 44 | 0.63 | 0.39 |  |  |  |  |  |  |
| F3 | Item 52 | 0.59 | 0.36 | 0.55 | 0.89 | F3–F4 | 0.67 | 0.02 | 0.63–0.71 |
|  | Item 9 | 0.65 | 0.34 |  |  | F3–F5 | 0.83 | 0.03 | 0.77–0.89 |
|  | Item 53 | 0.69 | 0.36 |  |  | F3–F6 | 0.65 | 0.02 | 0.61–0.69 |
|  | Item 54 | 0.62 | 0.32 |  |  | F3–F7 | 0.64 | 0.02 | 0.60–0.68 |
|  | Item 8 | 0.59 | 0.34 |  |  |  |  |  |  |
|  | Item 6 | 0.60 | 0.31 |  |  |  |  |  |  |
|  | Item 43 | 0.71 | 0.34 |  |  |  |  |  |  |
| F4 | Item 13 | 0.65 | 0.22 | 0.61 | 0.89 | F4–F5 | 0.63 | 0.02 | 0.59–0.67 |
|  | Item 12 | 0.70 | 0.18 |  |  | F4–F6 | 0.80 | 0.02 | 0.76–0.84 |
|  | Item 14 | 0.65 | 0.29 |  |  | F4–F7 | 0.73 | 0.02 | 0.69–0.77 |
|  | Item 19 | 0.51 | 0.38 |  |  |  |  |  |  |
|  | Item 24 | 0.63 | 0.19 |  |  |  |  |  |  |
| F5 | Item 56 | 0.74 | 0.18 | 0.67 | 0.89 | F5–F6 | 0.66 | 0.02 | 0.62–0.70 |
|  | Item 58 | 0.67 | 0.21 |  |  | F5–F7 | 0.67 | 0.02 | 0.63–0.71 |
|  | Item 59 | 0.68 | 0.29 |  |  |  |  |  |  |
|  | Item 57 | 0.71 | 0.27 |  |  |  |  |  |  |
| F6 | Item 29 | 0.60 | 0.16 | 0.66 | 0.88 | F6–F7 | 0.69 | 0.02 | 0.65–0.73 |
|  | Item 34 | 0.61 | 0.30 |  |  |  |  |  |  |
|  | Item 33 | 0.69 | 0.21 |  |  |  |  |  |  |
|  | Item 30 | 0.67 | 0.20 |  |  |  |  |  |  |
| F7 | Item 21 | 0.61 | 0.2 | 0.54 | 0.78 |  |  |  |  |
|  | Item 20 | 0.63 | 0.46 |  |  |  |  |  |  |
|  | Item 15 | 0.61 | 0.24 |  |  |  |  |  |  |

F1: Factor 1, Basic Microbiology; F2: Factor 2, Critical Thinking; F3: Factor 3, Communication and Patient Assessment; F4: Factor 4, Compliance with Infection Control Guidelines; F5: Factor 5, Education of Patient; F6: Factor 6, Infection Control Leadership; F7: Factor 7, Prevention of Occupational Exposure

FL, factor loading; SE, standard error; AVE index, average variance extracted index; CR, composite reliability
