# Supplementary material for: Development of an infection control competency scale for clinical nurses: an instrument design study
Source: BMC Nurs. 2024 Apr 19;23:250. doi: 10.1186/s12912-024-01904-1 (PMC11027540; doi:10.1186/s12912-024-01904-1)
Supplement: Supplementary file 2 — Supplementary Material 2 [file 12912_2024_1904_MOESM2_ESM.docx]

Appendix A. The ICCS-CN scale

| No. | No. Item | Strongly disagree | Disagree | Neutral | Agree | Strongly agree |
| --- | --- | --- | --- | --- | --- | --- |
| 1. | I am capable of explaining the results of antibiotic susceptibility tests. |  |  |  |  |  |
| 2. | I am capable of explaining the results of microbial culture tests. |  |  |  |  |  |
| 3. | I am capable of explaining the concept of multidrug-resistant bacteria (e.g. MRSA, VRE, CRE, etc.). |  |  |  |  |  |
| 4. | I am capable of explaining the difference between normal flora, colonisation, and infection. |  |  |  |  |  |
| 5. | When gathering information related to infections, I refer to reliable literature with confirmed guidelines or sources. |  |  |  |  |  |
| 6. | When referring to literature related to infection, I consult the latest publications within the past 5 years. |  |  |  |  |  |
| 7. | I have no difficulty in finding literature related to infection control. |  |  |  |  |  |
| 8. | I actively search for information on recent infectious disease outbreaks |  |  |  |  |  |
| 9. | I apply infection-related theories or evidence when performing infection control practice |  |  |  |  |  |
| 10. | I am knowledgeable about the antimicrobial spectrum of the antibiotics commonly used in our department and use them appropriately |  |  |  |  |  |
| 11. | I freely communicate with other healthcare professionals regarding matters related to infection control. |  |  |  |  |  |
| 12. | When assessing for symptoms of infection, I also consider atypical signs of infection (e.g. proteinuria, confusion in elderly patients) |  |  |  |  |  |
| 13. | I accurately document the patient’s infection risk, symptoms, reporting and actions and any changes in detail. |  |  |  |  |  |
| 14. | I share data or information related to infection control with department members. |  |  |  |  |  |
| 15. | I am capable of providing input on the re-evaluation of antibiotic administration based on test results or theoretical considerations |  |  |  |  |  |
| 16. | I am sensitive to noticing new symptoms of infection or changes in the infection status |  |  |  |  |  |
| 17. | When assessing the risk of infection, I consider potential risk factors |  |  |  |  |  |
| 18. | I perform hand hygiene as a routine and natural practice. |  |  |  |  |  |
| 19. | I perform hand hygiene at the appropriate times using the correct techniques |  |  |  |  |  |
| 20. | When administering medication via injection, I follow infection control guidelines by disinfecting the injection site before administering the medication |  |  |  |  |  |
| 21. | Isolated patients use dedicated medical equipment for isolated patients." |  |  |  |  |  |
| 22. | If I find a contaminated area, I do not leave it as is and remove it as soon as possible |  |  |  |  |  |
| 23. | When I provide infection-related education to the patient, I consider the age, education level, and situation of the patient (e.g., selecting appropriate terms, providing educational materials such as videos and documents, detailed explanations, etc.). |  |  |  |  |  |
| 24. | I can lead the patient to follow infection control precautions (e.g. personal hygiene, coughing after surgery, hand hygiene, adherence to isolation guidelines, etc.). |  |  |  |  |  |
| 25. | I understand the psychological state of patients infected with multidrug-resistant bacteria and help them make rational decisions (e.g. administration of antibiotics, cooperation in entering the isolation room, etc.). |  |  |  |  |  |
| 26. | I explain disease information, treatment procedures, and precautions based on evidence when a patient is infected. |  |  |  |  |  |
| 27. | I try to improve my behavior when receiving reasonable criticisms related to infection control from others. |  |  |  |  |  |
| 28. | I try to abide by the guidelines even in situations where infection control is difficult. |  |  |  |  |  |
| 29. | I can request work necessary for infection control from the person in charge of environmental management, such as a cleaning staff. |  |  |  |  |  |
| 30. | I strive to improve my weaknesses in knowledge, skills, and practices related to infection control. |  |  |  |  |  |
| 31. | I take steps to protect my skin wounds from infection. |  |  |  |  |  |
| 32. | I wear appropriate personal protective equipment and/or use tools when handling medical waste (e.g. not pressing down waste with feet, using tongs when handling sharps waste, etc.). |  |  |  |  |  |
| 33. | I wear personal protective equipment appropriate to the expected exposure type and infection transmission route when caring for the patient. |  |  |  |  |  |
